# Comparative Physiology of Menstruation

**Published:** 1883-10

**Authors:** Alfred Wiltshire

**Affiliations:** London


### ART. XXVIII.—THE COMPARATIVE PHYSIOLOGY OF MENSTRUATION.

BY ALFRED WILTSHIRE, M.D., F.R.C.P., LONDON.

*From the London Veterinary Journal.*

THE comparative method of study is, before all, the most trustworthy help towards the attainment of accurate knowledge, since, by its aid, a just appreciation of the relative value of facts may best be acquired. Accordingly, it merits employment whenever available, furnishing an efficient corrective of erroneous or disproportionate conceptions. It is valuable, also, inasmuch as it may afford clues to, or enlighten us, respecting, important correlations; while its influence, in checking and balancing our views and conclusions, is uniformly benificial. Acquaintance with the value of this method has led me to regard certain departments of medicine by its light, and among them the function of menstruation, in the elucidation of which it contributes important and suggestive information.

The illumination thrown by the comparative method upon certain biological problems ancillary to medicine is great; and the fervent, if not sanguine, hope is excited, that not only may similar enlightenment be vouchsafed in respect to recondite pathological problems, but that, through the pursuit of this instructive method, medicine, considered in its entirety one of the noblest studies, may, in process of time, be established upon foundations whence it may rise to a position not inferior to that accorded to other sciences.

The impression has long and widely prevailed, that the menstrual function is an attribute peculiar to the human female. Whether this be erroneous or not, will appear when inquiry is made into the manner in which the periodical activity of the reproductive organs is displayed or exercised in the females of the lower animals. Making due allowance for generic peculiarities, this function will be found to agree with others of the animal enconomy, in displaying harmonious subordination to the law of evolution. Inquired into on this basis, and in accordance with the comparative method, the evolution, of œstromenstruation is discovered to be orderly; and to correspond broadly with the evolution, anatomical and physiological, of the generative system of animals. In harmony with the universal result of biological research, the primitive indications of the function will be found to be but feebly marked. Gradually and progressively, however, the manifestations become more and more distinct, until, ultimately, the highest stage is attained in the human species. Thus traced step by step, it will be found that, from feeble and obscure beginnings in the lower creatures, the function emerges and develops until, in the highest mammals, it is unmistakably pronounced. It will also appear that, after its establishment at puberty—an epoch marking the assumption of the capacity for reproduction, and usually arriving only towards the time when the rate of growth is diminishing—its equilibrium is liable to flucuation, and even disturbance, owing to the extreme sensativeness of the reproductive system to vicissitudes of the environment, as Darwin and others have conclusively shown. It may be hoped that the laws governing this instability of equilibration will also be fully discovered, and, in process of time, admit of formulation, so to render them amenable to remedial modification. Pursued and investigated on such a basis, there is a prospect that the physiology of the function may be brought within the domain of science, and cease to be a wonderment and mystery. It may also be hoped that the discovery of the physiological laws governing it will furnish, not only a foundation for the establishment of a sound and intelligent system of therapeutics, for correcting aberrations and remedying deviations, but also a more reliable basis than at present exists for pathological knowledge. Mr. Herbert Spencer well says (*Data of Ethics*, p. 277):—" Pathological science depends for its advances on previous advances made by physiological science. The very conception of disordered action implies a preconception of well-ordered action. Before it can be decided that the heart is beating faster or slower than it should, its healthy rate of beating must be learnt; before the pulse can be recognised as too weak or too strong, its proper strength must be known; and so on throughout. Even the rudest and most empirical ideas of diseases presuppose ideas of the healthy states, from which they are deviations; and obviously the diagnosis of diseases can become scientific only as fast as there arises scientific knowledge of organic actions that are undiseased." The gain accruing from accurate physiological knowledge may thus be great, and ever growing.

So conspicuous a manifestation of sexual aptitude as a menstrual flux, whether sanguineous or not, is hardly to be looked for in the females of classes, below the mammalian, with which the study of the evolution of the catamenial function might appropriately commence, for "in the classes of birds, fishes, and oviparous reptiles, there is no uterus" (Laycock.) But there are generative canals, out of which, in the process of evolution, the uterus arises, just as does the bladder out of the renal excretory canals; being developed by the aggregation of muscular fibres, and differentiation of epithelial elements at certain parts (like the stomach, with its powerful muscular and glandular apparatus in the higher mammals, or the gizzard in the fowl); and it may be useful to trace their evolution, for, even in certain non-mammalian creatures, the excitement attending the active exercise of the reproductive function is occasionally displayed by an increased vascularity and pigmentation of certain structures, particularly about the genital orifices, which exhibit a kind of efflorescence at the periods of sexual excitement. For instance, according to Pouchet, "Guersant says that, at the epoch of the oviposit (in fishes), the orifice of their sexual apparatus swells, and is clothed with a red tint. Savants who, like Spangenberg, are occupied in studying the genital organs of birds, have recognised that they experience also at the time of the oviposit a manifest excitement." I am informed that the genitals of the parrot and pigeon tribes show this excitement. In other creatures, *e. g.*, amphibia, the orifices of the generative canals opening into the cloaca are generally increased in size at the breeding season. Reptiles also show increase in cutaneous secretions, especially of the odorous variety, in association with sexual activity. Laycock observes: "Many tortoises smell of musk, which probably proceeds from follicles connected with the cloaca. Several lizard, among others the iguana, have a row of small follicles with round orifices at the inner side of the thigh, which secrete, especially at the coupling season, an odorous fatty liquid;" and Mr. Darwin (*Descent of Man*, p. 352) says: "During the breeding season, the anal scent-glands of snakes are in active function, and so it is with the same glands in lizards." As we ascend in the scale of creation, we shall find that the genital apertures of the higher creatures present analogous phenomena, culminating in the highest even in an issue of blood.

Attention may here be directed, *en passant*, to the general relations of pigmentation to reproduction, which are striking, and, as Mr. Darwin has shown, are in birds remarkable. He says (*Descent of Man* p. 229): "Many birds acquire bright colors and other decorations in the breeding season alone;" and (p. 496) "certain ornamental appendages become enlarged, turgid, and brightly colored during the act of courtship." Even in insects, sexual pigmentation is often highly conspicuous; and Mr. Darwin (*Ibid.*, p. 265) says: "The sedentary annelids become duller-colored, according to M. Quartrefages, after the period of reproduction; and this, I presume, may be attributed to their less vigorous condition at that time." We may therefore derive instruction from the observation of the suggestive phenomena accompanying the active exercise of the reproductive functions, even in the lowest creatures.

Before the difference of sexes arises, both sexual elements (sperm and germ) exist, and are carried in the body of a single individual, though, curiously enough, the congress of two separate individuals, is in some cases necessary for fertilization, *e. g.*, in creatures which are not stationary.

Gegenbaur (*Elem. Comp. Anat.*, p. 54) says: "All those animals which unite in themselves both kinds of productive organs are known as Hermaphrodites. A separation of sexes is apparently foreshadowed in various forms, by the alternating activity of the organs, at the one time the egg-forming, and at another time the sperm-forming organ exercising its function. Hermaphroditism is the precursor of sexual differentiation...

A separation of the sexes effects the whole of the organism, for it produces a series of changes in each sex, which effect organs that had primitively little to do with the sexual function;" and Darwin remarks, "It has now been ascertained that, at a very early embryonic period, both sexes possess true male and female glands."

Before generative ducts arise, the generative products escape from the bodies of the lowest creatures by a cœlomic orifice, and occasionally, even in this primitive stage, some excitement is displayed around the aperture; but in the stage immediately above, when ducts begin to appear, imperfect though they be, we find that there is a remarkable constancy in the relation they bear to the renal excretory canals; that, in fact, the generative products, having no proper canals of their own, make use of those of the renal system, and these often display pigmentation at their orifices.

According to Gegenbaur (*Elem. Comp. Anat.*, p. 609), "The germ-glands are developed from the structures known as genital ridges. Sometimes more and sometimes less of this ridge is converted into the ovary or testis." These genital ridges are found in the abdominal cavity, the epithelial investment of which "retains its primitive character along a tract which corresponds to the rudiment of the primitive kidney longer than it does in other regions; and this epithelial layer may be distinguished as the germinal epithelium."

In the lowest forms, there are no generative canals. "Both sets of generative products are passed into the cœlom, whence they reach the exterior by the abdominal pore......In the Salmonidæ, the eggs are passed into the abdominal cavity, and are evacuated through the abdominal pore." Again (p. 53): "In their simplest condition the products of the reproductive glands merely break away from the spot where they are formed, and pass into the digestive sac, or into the body cavity, or even directly to the exterior. Gradully, however, ducts, which are often very complicated in character, are added on; it is probable that these ducts, are not primitively connected with the germinal glands. Where these ducts can be seen to have relation to other organs, these appear to be excretory organs, and have been altered so as to correspond to this function. It becomes a great question whether the excretory ducts of the reproductive matter are not in all cases excretory organs."

When, having passed beyond the primitive ductless stage, inquiry is made into the origin of the excretory ducts of the reproductive glands, we find that they are furnished by the primitive renal excretory apparatus (archinephron), the organs which eliminate the nitrogenous excreta from the body—organs distinctly derived from dermal glands (Gegenbaur, p. 46). The primary archinephric duct divides into two parts, so that there come to be two canals. "One commences at the anterior abdominal orifice of the primary duct, and has no further relations to the kidney. This is the Müllerian duct" (*Ibid.*, p. 64). Müller's duct becomes, in females, efferent, or oviducts, portions of which are ultimately differentiated into uteri. (The other archinepheric duct becomes the efferent duct of the kidney, or ureter.

And the late Mr. Balfour, in his admirable work on *Embryology*, shows how closely related the excretory and generative ducts are in the vertebrata.

The basis for the generative and urinary ducts is formed by the segmental duct, which is the duct of the pronephres. These, he says, "are the most primitive parts of the vertebrate excretory system." The meso-nephros, or Wolffian body, is formed of glandular canals, which open in the body cavity of the embryo. The segmental duct becomes, in many forms, divided longitudinally into two parts: one with segmental tubes, forming the Wolffian or mesonephric duct, the other the Müllerian duct.

The intimate relations thus indicated primitively between the urinary and generative organs, foreshadow a connection which persists even in the highest mammalia, a point remarked by Aristotle.

In the Amphibia, the Müllerian ducts form oviducts, opening separately into the cloaca. "It is generally increased in size at the breeding season; this results in its being thrown into a number of coils. In the oviparous species (Salamandra), the terminal portion performs the function of a uterus" (Gegenbaur, p. 612). A similar but more advanced condition obtains in the Sauropsida, the oviducts being large coiled canals, with mucous membrane set in longitudinal folds, which are most marked in the lower portion. This latter portion secretes the shell, the anterior part the albumen.

We find, then, that even in certain low forms, as in some of the foregoing, the orifices of the genital canals are apt to display pigmentary or vascular efflorescence at the breeding times. The stimulating influence of activity in the initiatory acts of reproduction upon pigmentation generally is remarkable and conspicuous throughout nearly the whole of the animal and vegetable world. The exquisite beauty of flowers is sexual, and in close alliance therewith is their perfume, often equally attractive.

The brilliant hues of many birds, fishes, etc., owe their existence to a sexual origin; and, as already remarked, the orifices, whence the germinal product escapes, are in some of the latter highly pigmented.

It is, however, with the Mammalia that our study of the comparative physiology of menstruation properly begins. They, as a class, are elevated above the other portions of the animal world by the endowment of a higher organization in many respects, and particularly by the possession of organs conferring upon them their distinctive appellation; organs which we shall find to exist in correlation with certain developments of other portions of the reproductive system, as well as of the general system.

Gegenbaur (p. 615) remarks: "In the Mammalia, the generative apparatus undergoes great metamorphoses, owing to the further development of various portions of the efferent ducts and the formation of a number of accessory organs. In the female apparatus, these are largely correlated with the relations that obtain between the embryo and the maternal organism. As this is least marked in the Monotremata, they undergo the least amount of modification, and have therefore direct relations to the lowest divisions of the Vertebrata, and especially to the Sauropsida. The oviducts open separately into a sinus urogenitalis, which communicates with the cloaca. The lower end of the oviduct, which is distinguished by the greater thickness of its muscular wall, forms an uterus; but this merely corresponds to the structures which likewise function as an uterus in any Anamia and Sauropsida."

With regard to the Monotremata, the lowest members of the mammalian series, but little information is available respecting the phenomena observable at their periods of sexual excitement. The class is now neither numerous nor widely distributed, and opportunities for inquiry have not been at my command.

Pouchet remarks of them: "Many of the lowest mammalia are ovoviviparous (*Ornithorhynchus paradoxus*, Blum.; *Echidna hystrix*, Cuv.), or produce only simple embryos (*Didelphys*), whilst all the others present the characters of most marked viviparity." They resemble birds in possessing a common cloaca, into which the generative, renal, and intestinal canals open. They have no vaginæ, and the uteri are rudimentary, affording no provision for the sustained growth and nourishment of the fœtus, which, like that of marsupials, is soon cast out.

In Marsupials (such as the kangaroo), the evolution of the sexual system is somewhat more advanced, there being no vaginæ, as the viginal canals open into the sinus urogenitalis. The upper portion of each different duct forms an oviduct, while the next and thicker-walled portion forms an uterus. Gegenbaur says: "Each of the two uteri opens by a papilliform process into a portion, which from the exterior appears to be common to them both, and which is formed by the union of the two Müllerian ducts. A curved vagina is given off from this on each side (Didelphys), or the commencement of the tube is replaced by the cæcal sac which is pushed out backwards, and is usually, though not always, divided internally by a medium partition; from this sac, the distinct 'vaginal canals' pass in a curved direction to the urogenital sinus (Halmaturus.)"

My inquiries into the manifestations of sexual aptitude exhibited by the females of Marsupials, like the kangaroo, tend, so far as they go, to support the hypothetical conclusion to which my researches had already led me, namely, that in accordance with their lowly position as mammals having inferiorly evoluted sexual organs, they would disply comparatively slight and inconspicuous local evidences of the rut or "heat."

Kangaroos are known to breed in this country; and although certain gestative phenomena are still shrouded in obscurity—*e, g.*, the mode of transference of the immature embryo to the marsupial sac, which, according to Owen, occurs in Macropus Major about thirty-eight days after impregnation—yet the times of "heat" have been recognized, and copulation witnessed, but only lately has any œstrual discharge been remarked. It must be remembered that in these creatures one orifice serves for the exit of fæces, urine, and generative products (in Marsupialia "there is even a common sphincter for the anus and urogenital orifice," Gegenbaur, p. 622), and that this, when closed, conceals in the cloaca the several corresponding apertures opening thereinto. Probably, therefore, whatever exudation attends the epochs of sexual excitement, if any there be, is hidden in the cloaca, unless, indeed, it should chance to be abundant, and that is not anticipated; neither, likewise, is it to be expected that a flux would partake of a distinctly sanguineous character, unless in so feeble and insignificant a degree as to be scarcely recognizable.

The most one appears warranted in expecting in these and allied lowly mammalia, is a mucous flux, perhaps feebly stained, with some increased odorousness, which, as in the higher mammals, is highly attractive and exciting to the males.

I have gratefully to acknowledge my indebtedness for information respecting the kangaroos at the Zoölogical Gardens to Mr. Bartlett, the able superintendent, who informs me that the Society's kangaroos display sexual excitement in September (which, he believes, corresponds in the southern hemisphere to our spring-time), and also in our spring month of April, the returning warmth of the season apparently stimulating them.

Until recently, neither Mr. Bartlett nor the keeper of the kangaroos had remarked any exudation or discharge from the cloacal orifice of the femals at the time of "heat;" but, since attention has been directed to this point at my request, the keeper informs me that he has observed a "mattery slimy" discharge, slightly tinged with a reddish color, at these times. He also informed me that, lately, prolonged copulation in a young female kangaroo, which had never bred, caused hæmorrhage, necessitating separation from the male. When impregnation has occurred, a close watch has been kept upon the females, in the hope of discovering the mode of transference of the embryo from the generative passages to the marsupium, but, hitherto, unsuccessfully. Mr. Bartlett tells me that something resembling blood and mucous has been seen to be conveyed by the female in her fore-paws from the cloaca to the marsupium, in which it was supposed the embryo might be entangled; and the keeper says that they sometimes find "hollow fleshy things" in the pens (fœtal envelopes); but I understand that no conclusive observations have yet been made. The issue of blood in question, small though it be, at what in these creatures is equivalent to the parturient act, suggests obvious analogies.

Above these grades, whose sexual systems and mode of reproduction are peculiar (*aplacentalia*), we come to a multitude of mammals of various kinds (*placentalia*), concerning some of which observation has taught us many interesting facts, though these are still too few to satisfy legitimate desire for information.

In all creatures there are periods of "heat" or sexual excitement, when both males and females are apt for procreation. These periods are marked by systematic excitement, and by local phenomena of a more or less conspicuous character. Among the mammalia, the times of heat display a seasonal periodicity; that is to say, they recur annually, biennially, quarterly, or at more frequent intervals, according as the conditions of existence or environments are favorable, or the reverse, and, apparently in some degree, according to the size of the creature, being for example, rare in the elephant. Yet in every creature seasonal periodicity, at longer or shorter intervals, and, as Darwin and Laycock have shown, this periodicity always partakes of an hebdomadal character. It is always some greater or lesser multiple of a weekly period.

In the lower animals in a wild or feral state, the aptitude for procreation is seasonal, recurring mostly at times when food and warmth are plentiful. Under normal circumstances, the earliest longing for sexual congress is promptly gratified; in the female, conception ensues, gestation proceeds in its appointed course, and until the genesial cycle has been completed by parturition, the "rut" or "heat," with its attendant desire for copulation does not ordinarily occur. But, impregnation failing from any cause (*e. g.*, absence of the male at the appropriate season of sexual appetite), we may inquire, do the symptoms of heat persist indefinitely? and, if not, what period elapses before they are again exhibited? Observation of wild animals, both in a state of nature and during captivity (when the latter does not interfere with reproduction), and, still better of domesticated animals, shows that, after a definite period of quiescence, œstruation, or the "rut," invariably recurs at epochs which strictly conform to some multiple of weeks. In highly-bred and well-cared-for domesticated animals, œstruation would probably be renewed periodically, until arrested by conception; for it is well known that domestication enormously enhances the capacity for reproduction, and renders that sustained which, under other conditions of environment, subsides until it is renewed by the seasonal awakening. It is probable that, in the wild state, in the absence of the male, the "heat," or stimulus to sexual desire, would, after a few periodical manifestations, die away, grow cold, and subside; remaining in abeyance until the return of the season with which it is primitively allied. This long rhythmed periodicity may be termed "seasonal," since it appears to be primarily allied with or dependent upon seasonal changes, as has been abundantly demonstrated by Darwin, Herbert Spencer, Laycock, and others. This seasonal periodicity of "heat" accounts for a corresponding periodicity in delivery, for, as is well known, many wild females bring forth their young at mild and favorable seasons, as spring, and not at inclement or unfructuous times. This is generally so in our domesticated animals when art does not interfere with their natural instincts; and, in truth, this primitive seasonal condition of the exercise of the generative function underlies the process of reproduction, even in the highest creatures. A trace of this seasonal influence is certainly still conspicuous in the greater tendency manifested by the human female to conceive at certain annual epochs; and it seems probable that this seasonal influence underlies the genesial function in all creatures, and is a relic and trace of a primitive or primordial condition governing reproduction.

We shall see that not only does woman most frequently begin their menstrual life in the summer months, but she brings forth her offspring more frequently in the spring than in other other seasons, just as the lower animals do. Many years ago, I concluded that every woman had a law peculiar to herself, which governed the times of her bringing forth (and conceiving); that, in truth, she was more prone to bring forth at certain epochs than at others; and subsequent researches have not only abundantly confirmed this surmise, but established the accuracy of the forecast. The evidence is given in other lectures.

The influence of civilization and domestication in expanding the reproductive powers is conspicuous. And yet the generative system, as Mr. Darwin has ably and conclusively shown, is highly sensitive to changes in the environment of the individual. Seemingly, the higher mental endowment of advanced human beings carries with it not only an augmented capacity for the reproduction of their own species, but also bestows a like advantage upon the creatures showing amenability to man's sway by flourishing under his dominion. Not alone does the human race increase, but man's flocks and herds multiply prodigiously. Would that proportional care were observed in the breeding and propagation of the human species, as, *e. g.*, is taken in that of animals of merely commercial value!

*(To be continued.)*

